# Potential natural products for the management of autism spectrum disorder

**DOI:** 10.1002/ibra.12050

**Published:** 2022-06-21

**Authors:** Punya Sachdeva, Intizaar Mehdi, Rohit Kaith, Faizan Ahmad, Md Sheeraz Anwar

**Affiliations:** ^1^ Amity Institute of Neuropsychology and Neurosciences Amity University Noida Uttar Pradesh India; ^2^ School of Studies in Neuroscience Jiwaji University Gwalior Madhya Pradesh India; ^3^ Department of Medical Elementology and Toxicology Jamia Hamdard University Delhi India; ^4^ Department of Psychology University of Campania Luigi Vanvitelli Caserta Italy

**Keywords:** autism, dietary supplement, natural products, neurodevelopmental disorder, neurotherapeutics

## Abstract

Autism in a broader sense is a neurodevelopmental disorder, which frequently occurs during early childhood and can last for a lifetime. This condition is primarily defined by difficulties with social engagement, with individuals displaying repetitive and stereotyped behaviors. Numerous neuroanatomical investigations on autistic children have revealed that their brains grow atypically, resulting in atypical neurogenesis, neuronal migration, maturation, differentiation, and degeneration. Special education programs, speech therapy, and occupational therapy have all been used to address autism‐related behavioral problems. While widely prescribed antidepressant drugs, antipsychotics, anticonvulsants, and stimulants have demonstrated response in autistic individuals. However, these medications do not fully reverse the core symptoms associated with autism spectrum disorder (ASD). The adverse reactions of ASD medicines and an increased risk of developing various other problems, such as obesity, dyslipidemia, diabetes mellitus, and thyroid disorders, prompted the researchers to investigate herbal medicines for the treatment of autistic individuals. Clinical trials are now being done to establish the efficacy of alternative techniques based on natural substances and to understand better the context in which they may be used to treat autism. This review of literature will look at crucial natural compounds derived from animals and plants that have shown promise as safe and effective autism treatment strategies.

## Graphical abstract

The figure explains that improvement like increased sound tolerance, better sleep pattern, better speech, improved social skills, and better eye contact and movement has been seen in patients with autism. An improvement in the symptoms can be observed by treating with natural products, such as camel's milk, luteolin, green tea, piperine, curcumin, cannabinoids, *Ginkgo biloba*, and *Bacopa monnieri*.

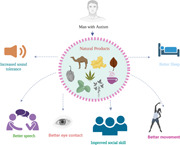

## 1 INTRODUCTION

In 1943, the term autism spectrum disorder (ASD) was coined by Leo Kanner, a child psychiatrist, when he came across 11 children who exhibited high detachment and an inability to build regular interactions with others. They were diagnosed with early infantile autism. Leo Kanner described those suffering from this illness as having an extreme desire for solitude and following a rigid schedule. They could easily spend hours distracting themselves with basic, repetitive activities and were easily agitated by the slightest break from their routine. Some autistic children could not speak or communicate.^1^ A year following Kanner's discovery, Dr Hans Asperger separately recorded four of his clients who shared comparable features but showed considerable intellectual ability in science and mathematics. Asperger mentioned to his patients that they possess autism despite this distinction.^2^ The symptoms frequently co‐occur with other comorbidities instead of appearing alone, including multiple psychiatric disorders, such as obsessive‐compulsive disorder, attention deficit hyperactive disorder and gastrointestinal conditions, and feeding disorders. In ASD, the term “spectrum” refers to the broad range of intensity of the symptoms, starting with minimally to seriously impaired autistic individuals who require long‐term expert support, frequently observed in affected children. ASD was reported to impact around 1%–2% of the overall population.^3^ In 2010, the worldwide prevalence of ASD was 7.6 per 1000 or 1 in 132 persons.^4^ From the late 1990s, ASD frequency has increased dramatically over time.^5^ Numerous studies have claimed that changing case definitions and improved awareness account for the apparent increase.^6^ Considering the rise in prevalence and detection of side effects of medicines, scientists have worked to bring out some of the natural products having the potential role in managing the clinical manifestations in individuals with autism. Thus, this literature review elaborates on some of the significant and effective naturally occurring products for managing the symptoms associated with ASD.

## 2 ENVIRONMENT AS A CAUSE FOR ASD

Vaccination, maternal smoking, thimerosal exposure, and, most likely, assisted reproductive technologies are all unrelated to the risk of ASD, according to current evidence. On the other hand, older parents are linked to a higher incidence of ASD. Birth problems linked to trauma, ischemia, and hypoxia have also been linked to ASD, although other pregnancy‐related variables, such as maternal obesity, diabetes, and caesarian section have demonstrated a less (but still substantial) link to ASD risk. The design of toxic element research has severely limited their findings; however, there is sufficient evidence for a link between specific heavy metals (most notably inorganic mercury and lead) and ASD to suggest further investigation.^7^ Maximized possibility of autism is concerned with the exposure of the fetus to air pollution, poisons (thalidomide, retinoic acid, and valproic acid), and particulates.^8^ Unhealthy lifestyle, prenatal stress, diet, and family history where the family members have suffered from several infectious diseases are all variables that contribute to an autistic newborn's behavioral abnormalities.^9^ Autism is associated with perinatal conditions such as significantly low birth weight and hypoxia during birth or premature delivery.^10^ Genetic variability may also increase due to environmental factors, which have been linked to enzymatic impairments in autism. Gene−environment interactions are complicated, and their mechanisms remain unknown at the molecular level.^10^

### 2.1 Genetic makeup of ASD

The intricacy of ASD and its range of clinical manifestations may be explained by gene−gene interaction, as well as the influence of epigenetics, that is, exposure to environment‐associated modifiers or stressors that alter the expression of the gene.^11^, ^12^ Additionally, ASD has been linked to polygenic polymorphisms, single nucleotide variants, copy number variants, and uncommon inherited variants.^13^, ^14^ The uneven sex distribution, greater incidence in siblings, more concordance in monozygotic twins, and higher risk of ASD with more relatedness all imply a strong influence of genes on the occurrence of ASD.^14^, ^15^, ^16^ Numerous studies have presented that male‐to‐male transmission occurs in several families, hence eliminating X‐linkage as the exclusive mode of inheritance.^17^ Additionally, it was discovered that the frequency of ASD among siblings is more than the prevalence rate in the general population.^18^ The relationship between clinical characteristics and particular genetic profiles is still being investigated.^19^, ^20^

## 3 CHANGES NOTICED IN AUTISTIC BRAIN

Neurobiological research in ASD patients, including neuroimaging, electrophysiology, and autopsy, has suggested that brain abnormalities, particularly aberrant neural connections, have a crucial significance in the occurrence of ASD.^21^, ^22^ Moreover, ASD children's heads perhaps expand more rapidly throughout infancy, and their overall brain size may be larger.^23^ While comparing people having ASD with non‐ASD shows a significant difference in total gray and white matter volumes in some regions of the brain, altered brain neuromodulator concentrations, changed neural circuit anatomy, distorted gyral and sulcal anatomy, changed lateralization of the brain, and altered structure and anatomic organization of the cortex.^24^ Additionally, aberrant neuronal differentiation during prenatal development appears to cause cortical abnormalities.^25^ Furthermore, in comparison to people without ASD, patients with ASD use different neural pathways for cognitive processes, and specific brain regions process information during activities that require interaction in society (e.g., eye gazing, faces, speech).^26^

## 4 CROSSTALK BETWEEN DIETARY SUPPLEMENT DEFICIENCY AND ASD

Experiments and population‐based investigations have demonstrated that the pathogenic alterations associated with ASD appear to begin during fetal development. The behavioral and neurological aspects of ASD in the fetus have been hypothesized to be acquired due to maternal metabolic disorders. Kawicka et al. previously demonstrated that metabolic problems, such as obesity and diabetes, during pregnancy might be one of the factors that fetus develop ASD.^27^ Appropriate nutritional intake is necessary for brain growth and maturation during pregnancy.^28^ Certain associative measures of children's nutritional and dietary status have suggested that a deficiency of some of the supplements, vitamins, and minerals, such as pyridoxine (vitamin B6), magnesium, calcium, folic acid (vitamin B9), omega‐3 fatty acids, potassium, iron, cholecalciferol (vitamin D), tocopherols (vitamin E), and zinc may be potential risk factors for ASD.^29^ Additionally, abnormally high amounts of copper, folic acid, iron, and calcium have impaired zinc absorption.^30^ Pregnant women who use excessive calcium and iron‐rich supplements may have a zinc absorption deficit. Zinc and iron deficiency appear to be a part of alteration in the expression of genes involved in neuroplasticity and neurogenesis during the prenatal period, including BDNF, SDF‐1, CamKIIa, and PSD‐95. Certain pharmacological drugs, such as angiotensin‐converting enzyme (ACE) inhibitors, which are extensively used to treat hypertension, may also cause a drop in blood zinc levels.^31^ Additionally, folic acid is required for normal erythropoiesis and neural tube formation.^32^ While some studies have indicated that folic acid intake effectively treats ASD, problems in folate metabolism and folic acid overload during pregnancy have been associated with the development of ASD symptoms in progenies.^33^ Vitamin D is found in less amounts in the diet; it is generally absorbed through skin exposure to sunlight.^34^ Vitamin D deficiency caused by certain environmental conditions, especially the weather, has been linked to ASD.^35^ Vitamin E has long been recognized as a potent antioxidant protecting the body from oxidative stress. According to research, children with vitamin E deficiency frequently exhibit autistic‐like behavioral abnormalities.^36^ The polyunsaturated fatty acids (omega‐3 and omega‐6 fatty acids) appear critical for brain development and neuroplasticity regulation. Consumption of omega fatty acids appears to decrease due to lifestyle changes. As a result, omega fatty acid deficiency has been identified as a risk factor for ASD.^37^ Gastrointestinal (GI) issues have been recognized as a frequent symptom in individuals.^38^

On the one hand, changes in the gut microbiome and gastrointestinal illnesses may impair the digestion of dietary supplements, resulting in vitamin, mineral, and other essential nutrient deficiencies. However, it may disrupt the gut−brain axis.^39^ It is well established that the gut−brain axis influences brain development and behavior via modulation of neurogenesis, neuroplasticity, neuroendocrine, and neuroimmune activities.^40^ Dairy and gluten‐containing diets significantly affect the equilibrium of the gut microbial environment, impairing the gut−brain axis and further impairing neuronal processes.^41^ Thus, disruption of the gut−brain axis, which is frequently observed in individuals with aberrant behavioral patterns, may also play a significant role in the development of ASD.^42^ Additionally, food allergens,^42^ and the accumulation of some toxic metals, such as cadmium, arsenic, and mercury, have been implicated in the development of ASD.^43^

## 5 CURRENT THERAPEUTIC INTERVENTIONS FOR THE TREATMENT AND MANAGEMENT OF AUTISM

While some psychosocial therapies are helpful, there is no effective therapeutic plan for ASD. Affected individuals have a wide variety of symptoms that vary significantly.^44^ Treatment approaches are customized for every patient. Specialized training, educational programmers, and behavioral therapies may aid in the development of job skills, self‐care, and maturity, while drugs may help alleviate anxiety and irritation.^45^ Applied behavior analysis is a beneficial intervention that relies on unique one‐on‐one training assignments based on behaviorist principles of reward, stimulus, and response.^46^ Discrete trial training teaches essential skills like imitation, attention, and compliance through a somewhat different technique. In autistic persons, pivotal response training promotes self‐management and social bonding. Anticonvulsants, stimulants, antidepressants, and antipsychotics, such as risperidone or aripiprazole, are typically provided to diagnosed children.^47^ However, the long‐term consequences of such medications must be thoroughly researched, as each individual reacts differently to them.^48^

## 6 ROLE OF DIFFERENT POTENTIAL NATURAL PRODUCTS IN ASD

**Figure 1:**
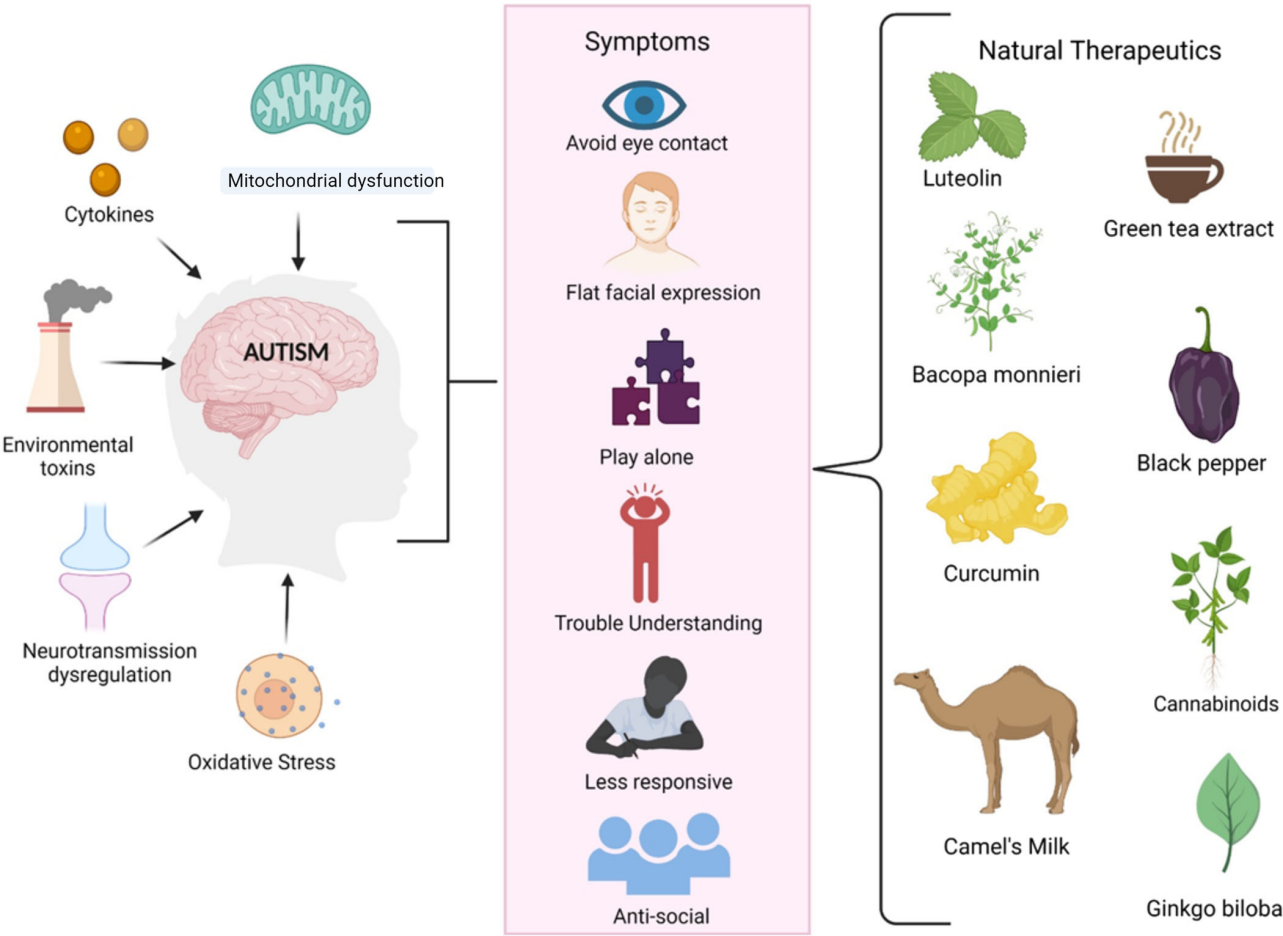
The figure shows the potential risk factor associated with ASD, such as the release of cytokines, mitochondrial dysfunction, oxidative stress, neurotransmission dysregulation, and environmental toxins. It has been seen that the release of pro‐inflammatory cytokines, which causes inflammation, influences the development of ASD. The elevated levels of oxidative stress lead to mitochondrial dysfunction through disrupted energy regulation. Impaired mitochondrial function, neurotransmission, and prolonged exposure to environmental toxins are linked with abnormal brain development, causing ASD. ASD, autism spectrum disorder. [Color figure can be viewed at wileyonlinelibrary.com]

The requirement for safe and effective drugs for the efficient management of autism has resulted in exploring a variety of natural plant‐based products with therapeutic possibilities. Effective herbal treatments may reduce clinical manifestations with fewer side effects.^47^ The causes, symptoms, and potent natural products for the management of ASD are shown in Figure 1. Table 1 shows the effect of natural products on animal models and Table 2 shows the effect of natural products on children and adults with autism.

**Table 1:** Effect of natural products on animal model

Natural product	Nonclinical model name and method	Method	Result	References
Luteolin	Murine model	Valproic acid induced mouse	Improved nonsocial and social behavior	[49]
Green tea extract	Young mice (both male and female)	Dose: Valproate (400 mg/kg subcutaneously) was given to newborns on postnatal Day 14. Green tea extract (75 and 300 mg/kg) was given to newborns up to postnatal Day 40.	Neuronal cytoprotective effect and behavioral improvement at 300 mg/kg of green tea extract	[50]
Curcumin	Male black and Tan Brachyury (BTBR) mice	CUR (25,50, and 100 mg/kg, i.p)	Alleviates ASD‐like symptoms in BTBR mice and mitigates oxidative stress	[51]
Curcumin	Forty neonatal male Western Albino rats	Four rat groups, third group received valproic acid with curcumin, fourth group only curcumin	Improved delayed maturation and abnormal weight, corrected dysfunction of IL‐6, CYP450, glutamate, and oxidized glutathione	[52]
Piperine	BALB/c mice 13 days, 3 males 3 females	Five groups, behavioral test up to PND 40	Mice killed, improved behavioral alterations, lowered oxidative stress markers	[53]
*Bacopa monnieri*	12.5 days female pregnant rats	Control and valproic acid (600 mg/kg i.p)‐treated groups	Animals killed but ameliorate autistic symptoms	[54]

**Table 2:** Effect of natural products in children and adults with ASD

Natural product	ASD (children or adults)	Outcomes	References
Luteolin	Children	Thirty‐seven children were given luteolin for 4 months, which shows 10% improvement in speech, 25% improvement in social skills, 50% improvement in eye contact, and 75% improvement in gastrointestinal symptoms	[55]
Luteolin	Children	After 26 weeks of treatment with luteolin, TNF and IL‐6 levels reduced, which finally improved behavior	[56]
Luteolin	Children	Fifty children were given one capsule having 10 kg of weight per day along with the food and this dose reduced almost all symptoms with no major adverse effect	[57]
Cannabidiol	Adults	Thirty‐four adult men in which 50% are diagnosed with autism were given 600 mg cannabidiol, which alters the fractional amplitude of low‐frequency fluctuations	[58]
Cannabidiol	Children and adults (both)	One hundred and fifty participants from the age of 5−21 years were given whole plant extract, which shows 49% improvement in behavior with no major adverse effect	[59]
Camel milk	Children	500 ml camel's milk was given to around 45 children on daily basis for 2 weeks, the serum level of activation‐regulated chemokines decreased and the childhood autism rating scale score improved.	[60]

### 6.1 Luteolin for ASD

Microglia are a form of macrophage found in the CNS that perform comprehensive scanning and activation in response to stressors like damage, disease, or infection.^61^ Their activation is also associated with CNS inflammatory responses.^62^ Autism is connected with maternal immunological stimulation and the consequent microglial dysfunction in the development of the brain.^63^ Numerous etiological ideas with varying degrees of evidence suggest that targeting microglial activation to modulate these inflammatory cascades may benefit the treatment of autism.^64^, ^65^ Luteolin is a flavonoid that occurs naturally in food plants. Lutein therapy effectively inhibited the increase in glial fibrillary acidic protein (GFAP) in astrocytes induced by IL‐6 in a cell‐based human maternal immunological activation model.^65^ GFAP is typically overexpressed in proliferating glial scars.^65^ A significant decrease in the phosphorylated transcription factor STAT3 was observed.^65^ Excessive phosphorylation of STAT3 indicates increased cytokine and growth factor activity, frequently resulting in inflammation. Additionally, luteolin treatment decreases TBR1‐ and CTIP2‐positive cells.^66^ The expression of TBR1 and CTIP2 is required for proper cortical development during the earliest phases.^67^ Bertolino et al. demonstrated that the flavonoid luteolin combined with the fatty acid palmitoylethanolamide was neuroprotective and anti‐inflammatory. Neuroinflammation is one of the characteristic features of autism; elevated levels of interleukin‐6 (IL‐6) and tumor necrosis factor (TNF) are also detected in the serum of affected individuals.^68^ However, autistic youngsters who took a luteolin dietary supplement regularly demonstrated enhanced social bonding and behavior. Likewise, serum levels of IL‐6, TNF, and other cytokines significantly decreased with luteolin consumption.^56^

### 6.2 Green tea (*Camellia sinensis*) for ASD

Increased oxidative stress has been associated with autism development.^43^ Children with autism exhibit higher levels of lipid peroxidation, significant antioxidant serum proteins, altered glutathione status, and levels of critical antioxidant enzymes, such as superoxide dismutase, glutathione peroxidase, and catalase.^69^ *C. sinensis* contains a significant amount of caffeine, polyphenol, and flavonoids, with well‐established antioxidant properties. Experiments have demonstrated that green tea has many good health impacts.^70^ Flavonoids may cross the blood−brain barrier and possess a range of neuroprotective properties.^71^ The daily ingestion of green tea extract (75−300 mg/kg) is recommended for the production of neuroprotective effects in the brain.^50^ The bioactive components of green tea have been shown to impact the level of neurotransmitters in the brain directly, most importantly dopamine and serotonin in specific brain areas.^72^ l‐theanine, an amino acid found in tea, has antistress qualities and increases long‐term potentiation in an NMDA‐independent way; hence, it helps in improving memory.^72^ Autism is characterized by a decline in the functioning of Purkinje cells in the cerebellar region.^73^ Histological findings of naive mouse models who received 300 mg/kg of green tea extract consistently exhibited progressive regeneration of the Purkinje layer and cells, showing that green tea extract may well have neuroprotective qualities to treat autism. Moreover, green tea's cytoprotective activity on brain cells has been established, and it may be useful in managing symptoms of autism through early dietary intervention.^74^

### 6.3 Piperine for ASD

Piperine, chemically an *N*‐acylpiperidine, is the primary alkaloid extracted from black pepper and long pepper. The chemical can activate pain‐sensing nerve cells' heat and acidity‐sensing ion channels. Specifically, they are called nociceptors.^75^ It has a vital effect on the nervous system. Historically, it has been used widely in treating epileptic disorders, exhibits significant antioxidative properties, and helps in memory enhancement and cognition.^76^ Piperine pretreatment protected cultured hippocampus neurons against cell viability loss caused by a glutamatergic increase. The mechanism through which it operates action has been linked to the control of Ca2+ ion entry.^77^ Twenty mg/kg of sodium valproate was used experimentally to treat autistic Balb/C mice, after which they were evaluated behaviorally, histopathologically, and biochemically on postnatal Day 14. The piperine can elicit beneficial effects, as evidenced by its antioxidant activity, cognitive enhancement, and neuroprotective characteristics.^53^ Additionally, the chemical has anxiolytic properties, for which it acts as an antistress and relaxing medicine. Thus, clinical studies, including piperine research, are going toward elucidating its potential benefits for autistic children.^76^

### 6.4 Curcumin for ASD

Curcumin is the primary curcuminoid found in turmeric (*Curcuma longa*), a spice known for its neuroprotective qualities. It has been shown to target several signaling pathways inside the cell and play a role in controlling nitrosative or oxidative stress, mitochondrial function, and protein aggregation.^78^ Curcumin possesses a broad spectrum of anti‐inflammatory properties and can quickly cross the blood−brain barrier.^79^ It was discovered in a study that curcumin supplements have been shown to significantly increase the concentration of antioxidant enzymes.^80^ Curcumin at a dose of up to 200 mg/kg given to male Sprague–Dawley rats exhibiting autistic phenotypes has been shown to reduce oxidative stress, mitochondrial defect, tumor necrosis factor (TNF‐) release, and matrix degradation metalloproteinases. Thus, it has been observed that curcumin acts as a neuro‐psycho‐pharmacotherapeutic substance in treating ASD.^81^ As a direct result, curcumin can lower numerous inflammatory indicators in various disorders and has consistently exhibited antioxidant radical scavenging activity in vitro and in vivo.^82^ Increased synaptic plasticity, which results from regular consumption of curcumin in the diet, has been shown to improve cognition. However, there are no compelling data on clinical studies demonstrating the efficacy of clinical experiments on humans; evidence supporting curcumin's neuroprotective properties is enough for it to be used in upcoming autism research and additionally linked illnesses.^83^

### 6.5 Cannabinoids for ASD

Cannabis is currently being studied medically for various neurological illnesses, with success shown with its use.^84^ When used in sufficient amounts, tetrahydrocannabinol (THC), the phytocannabinoid that is the primary psychoactive component of *Cannabis sativa*, can worsen various neurological disorders. The study done by Salgado et al.^85^ states that cannabidiol (CBD) use can be efficacious in decreasing autistic behavior. The substance has an impact on immunomodulation, antioxidant defense, and neuroprotection and offers promising therapeutic alternatives with negligible or no side effects.^86^ Additionally, cannabidivarin (CBDV) has been demonstrated a favorable potential for ameliorating behavioral changes, and clinical trials with this chemical demonstrated significant improvement in autistic patients.^87^

Moreover, an additional 12 weeks of CBDV at a dose of 10 mg/kg/day has been approved as an assessment to ensure that it is tolerable and safe.^88^ The endogenous cannabinoid (EC) system is a crucial neuromodulatory mechanism. It can regulate emotional reactions and behavioral reactivity to a desirable degree of interpersonal communication. In most cases, ASD patients' EC systems are found to be compromised.^88^ Endogenous substances such as signaling chemicals produced from arachidonic acid and related enzymes can bind to and activate EC receptors, resulting in increased RNA and protein levels.^89^ But, when this mechanism fails, it disrupts normal metabolic pathways, resulting in neuroinflammation. Therefore, activating the EC system with natural cannabis phytoproducts may be beneficial in modulating the immunological responses, possess antioxidant properties, and aid in ameliorating the autism spectrum's symptoms.^88^

### 6.6 *Ginkgo biloba* for ASD

The standardized extract of *G. biloba* leaves contains around 24% flavone glycosides (mostly quercetin, kaempferol, and isorhamnetin) and 6% terpene lactones (2.8%–3.4% ginkgolides A, B, and C, respectively and 2.6%–3.2% bilobalide).^89^ Nearly 0.8% and 0.1%, respectively, were ginkgolide B and bilobalide. Additionally, proanthocyanidins, glucose, and organic acids are present, as well as rhamnose, d‐glucaric acid, and ginkgolic acid.^90^ The presence of terpenoids, organic acids, and flavonoids in the extract contributes to its efficacy. It protects against ischemic stroke, Parkinson's disease, and Alzheimer's disease.^91^ In an observational study, 100 mg/kg of *G. biloba* extract taken twice daily was beneficial in improving autistic people's symptoms and odd behavior. The extract has been demonstrated to resolve behavioral problems effectively; impatience, hyperactivity, poor eye contact, and improper speech are all characteristics of autism.^92^ *G. biloba* extract is used to treat autism with other drugs. The treatment group reported fewer adverse events compared to the placebo group. There is a knowledge gap about the pharmacokinetics and bioavailability of medicines in the CNS. More research is required to evaluate the efficiency of *G. biloba* in the treatment of neurological diseases, including autism.^93^

### 6.7 *Bacopa monnieri* for ASD

Bacosides are widely utilized medicinally by the tribes of India and are the primary bioactive constituents of *B. monnieri* (L.) West.^94^ This herb has long been recognized for its intellect‐ and cognition‐enhancing effects and its nerve tonic abilities.^95^ *B. monnieri*'s pharmacological activities have been linked to its alkaloids, saponins, and sterols constituents.^96^ *B. monnieri* considerably reduced behavioral changes in a BTBR T+ tf/J mouse model of oxidative stress, decreased pain threshold, normalized locomotor deficits, and anxiety autism model. The increased locomotive activity was clarified to *B. monnieri*'s antianxiety qualities and its capacity to reduce glutamate accumulation and restore the architecture of the cerebellum.^54^

### 6.8 Camel milk for ASD

The use of camel milk has lately been revealed to have potential treatment benefits for several ailments.^97^ In individuals with ASD, it was related to decreased plasma GSH and cysteine levels and has been demonstrated to have a favorable effect on the behavior. Autistic children showed significant improvements in the scale for evaluating autism in childhood (CARS) after camel milk consumption. Camel milk is unique in its composition and cannot be found in other ruminants' milk. In comparison to the udder of a cow, camel's milk has a greater concentration of elements (calcium, iron, magnesium, copper, zinc, and potassium) and vitamins (A, B2, E, and C), less fat, cholesterol, salt, and lactose.^98^

Additionally, it lacks beta‐lactoglobulin and beta‐casein, two critical active ingredients found in cow's milk intolerance. Camel milk has several protective proteins and enzymes that are antibacterial, antiviral, and immunologic.^98^ Immunoglobulins, lysozymes, lactoperoxidase, *N*‐acetylglucosaminidase, and peptidoglycan recognition protein are all crucial peptidoglycan‐recognition proteins for preventing and curing food allergies.^99^ Camel milk owes its origins to its anti‐inflammatory proteins, hypoallergenic properties, and smaller nanobodies.^97^ It can alleviate specific main autistic symptoms due to its hypoallergenic qualities and antibodies identical to those found in antibodies against humans.^100^ Nanobodies are present in milk. Due to their small size, camel milk nanobodies exhibit unique structural properties, including enhanced tissue penetration,^101^ and the ability to detect not immediately apparent epitopes. These qualities may aid in infection prevention and may confer extra advantages—the immune system's strength.^97^

**Figure 2:**
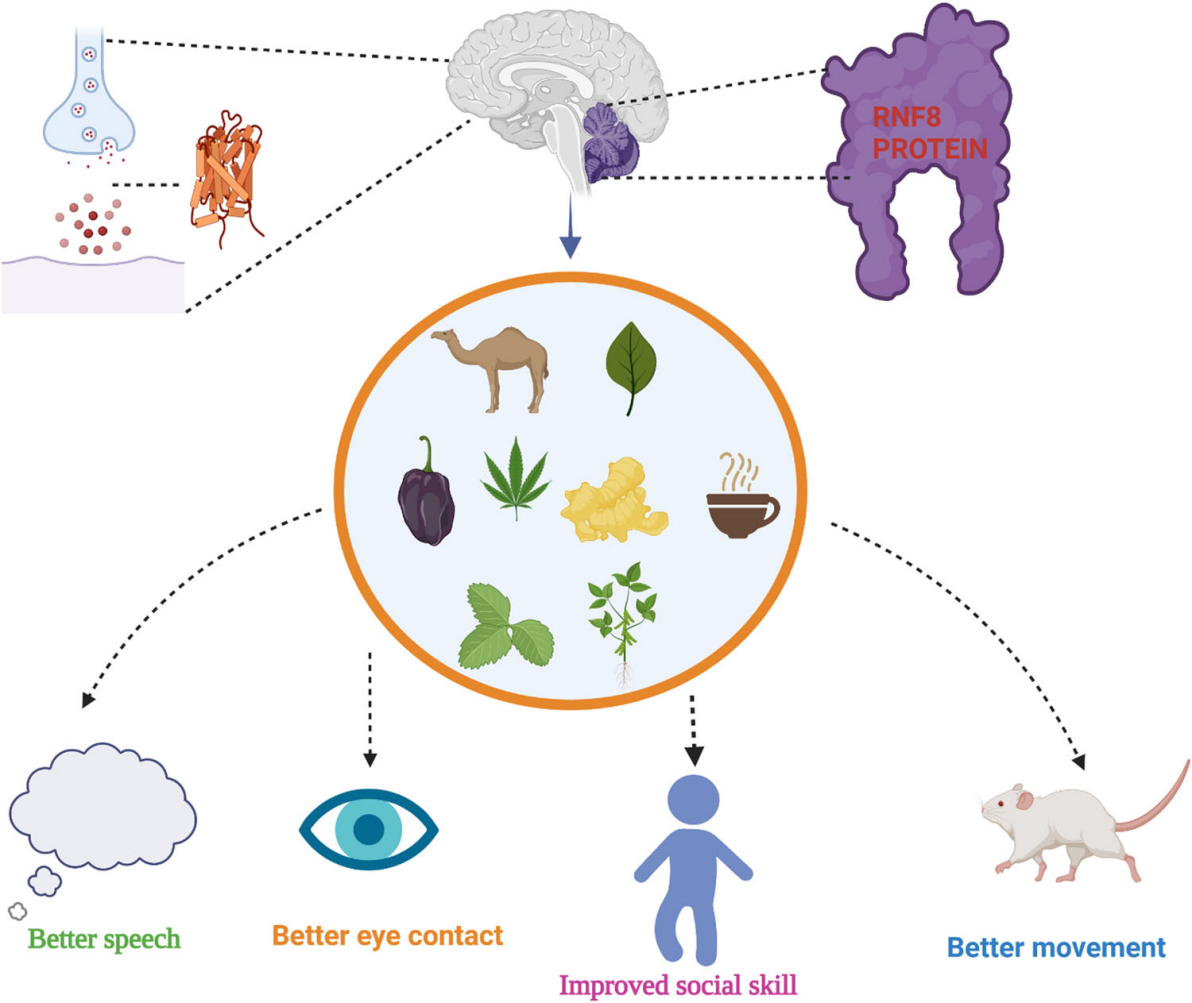
Autism affects the cerebellum, marked by blue color, and in the cerebellum, RNF8 protein is found. It has been seen that the absence of the RNF8 gene leads to impaired learning skills. GABA neurotransmitter is involved in autism, which natural products can balance. In preclinical and clinical trials reduced the negative symptoms and showed positive responses in mice (movement) and better speech, eye contact, and improved social skills in children and adults. [Color figure can be viewed at wileyonlinelibrary.com]

Furthermore, the structure of camel milk nanobodies is strikingly similar to that of human immunoglobulins (IgG3). This reveals that camel antibodies are identical to human antibodies. The unique composition of camel milk has been demonstrated to positively impact the condition of children with ASD by increasing superoxide dismutase levels and myeloperoxidase levels, plasma GSH levels, and oxidative stress. According to some studies, camel milk was utilized for 2 weeks as a potential treatment approach. There are significant differences in CARS, SRS, and ATEC scores among individuals with ASD.^97^ The trial's findings have shown that antioxidant enzymes and nonenzymatic antioxidant substances found in camel milk may have a vital component in the process of normalizing ASD behaviors. Tests on a broader scale that focuses on the dosage of the camel milk samples are necessary to look into its effect on oxidative stress markers and hence its antioxidant properties in ASD treatment.^97^ Figure 2 shows the impact of natural products on the symptoms associated with ASD.

## 7 FUTURE DIRECTION AND CONCLUSION

Natural products have demonstrated a plausible therapy for various conditions, including neurodevelopmental disorders such as autism. Numerous years of study have determined that the advantages and negative impacts of the above‐mentioned natural products have not been identified, established, or recommended in the near future. These naturally occurring chemicals discovered as possible therapeutic candidates can be used as chemical models or templates to synthesize or modify novel compounds for the treatment of autism. Plant resources, in particular, have the potential to be immensely valuable. There are FDA‐approved medications to address autistic persons' behavioral issues. However, the core symptoms of these drugs are not treated. Over half of patients get psychoactive drugs or anticonvulsants, particularly synthetic antidepressants, stimulants, and antipsychotics, all of which have a variety of harmful consequences when used indefinitely. While advanced learning approaches and alternative therapies are accessible nowadays, herbal medications remain a dependable option. All that remains is to determine their efficacy against autism. Enduring and unshakable, as evidenced by further investigations.

## AUTHOR CONTRIBUTIONS

**Punya Sachdeva**: Conceptualization; writing; editing; drawing figures; and reviewing. **Intizaar Mehdi**: Writing; editing; and reviewing. **Rohit Kaith**: Writing; editing; reviewing. **Faizan Ahmad**: Drawing figures and tables. **Md Sheeraz Anwar**: Reviewing; editing.

## CONFLICT OF INTEREST

The authors declare no conflict of interest.

## ETHICS STATEMENT

Not applicable.

## Data Availability

Data generated during the study are mentioned in the paper.

## References

[1] Kanner L . Etude de l'évolution de onze enfants autistes initialement rapportée en 1943 [Follow‐up study of eleven autistic children originally reported in 1943. 1971]. Psychiatr Enfant. 1995;38(2):421‐461. 8657796

[2] Dell'Osso L , Luche RD , Gesi C , Moroni I , Carmassi C , Maj M . From Asperger's *Autistischen Psychopathen* to DSM‐5 autism spectrum disorder and beyond: a subthreshold autism spectrum model. Clin Pract Epidemiol Ment Health. 2016;12:120‐131. 10.2174/1745017901612010120 27867417 PMC5095893

[3] Elsabbagh M , Divan G , Koh YJ , et al. Global prevalence of autism and other pervasive developmental disorders. Autism Res. 2012;5(3):160‐179. 10.1002/aur.239 22495912 PMC3763210

[4] Baxter AJ , Brugha TS , Erskine HE , Scheurer RW , Vos T , Scott JG . The epidemiology and global burden of autism spectrum disorders. Psychol Med. 2015;45(3):601‐613. 10.1017/S003329171400172X 25108395

[5] Kogan MD , Vladutiu CJ , Schieve LA , et al. The prevalence of parent‐reported autism spectrum disorder among US children. Pediatrics. 2018;142(6):e20174161. 10.1542/peds.2017-4161 30478241 PMC6317762

[6] Fombonne E . Epidemiology of pervasive developmental disorders. Pediatr Res. 2009;65(6):591‐598. 10.1203/PDR.0b013e31819e7203 19218885

[7] Modabbernia A , Velthorst E , Reichenberg A . Environmental risk factors for autism: an evidence‐based review of systematic reviews and meta‐analyses. Mol Autism. 2017;8:13. 10.1186/s13229-017-0121-4 28331572 PMC5356236

[8] Kinney DK , Munir KM , Crowley DJ , Miller AM . Prenatal stress and risk for autism. Neurosci Biobehav Rev. 2008;32(8):1519‐1532. 10.1016/j.neubiorev.2008.06.004 18598714 PMC2632594

[9] Lyall K , Schmidt RJ , Hertz‐Picciotto I . Maternal lifestyle and environmental risk factors for autism spectrum disorders. Int J Epidemiol. 2014;43(2):443‐464. 10.1093/ije/dyt282 24518932 PMC3997376

[10] Zachariah SM , Oommen SP , Koshy B . Clinical features and diagnosis of autism spectrum disorder in children. Curr Med Issues. 2017;15:6‐16.

[11] Muhle R , Trentacoste SV , Rapin I . The genetics of autism. Pediatrics. 2004;113(5):e472‐e486. 10.1542/peds.113.5.e472 15121991

[12] Bacchelli E , Maestrini E . Autism spectrum disorders: molecular genetic advances. Am J Med Genet, Part C. 2006;142C(1):13‐23. 10.1002/ajmg.c.30078 16419096

[13] Robinson EB , Neale BM , Hyman SE . Genetic research in autism spectrum disorders. Curr Opin Pediatr. 2015;27(6):685‐691. 10.1097/MOP.0000000000000278 26371945 PMC4650984

[14] Sandin S , Lichtenstein P , Kuja‐Halkola R , Hultman C , Larsson H , Reichenberg A . The heritability of autism spectrum disorder. JAMA. 2017;318(12):1182‐1184. 10.1001/jama.2017.12141 28973605 PMC5818813

[15] Hallmayer J , Cleveland S , Torres A , et al. Genetic heritability and shared environmental factors among twin pairs with autism. Arch Gen Psychiatry. 2011;68(11):1095‐1102. 10.1001/archgenpsychiatry.2011.76 21727249 PMC4440679

[16] Rosenberg RE , Law JK , Yenokyan G , McGready J , Kaufmann WE , Law PA . Characteristics and concordance of autism spectrum disorders among 277 twin pairs. Arch Pediatr Adolesc Med. 2009;163(10):907‐914. 10.1001/archpediatrics.2009.98 19805709

[17] Cheng Y , Qin G , Dai X , Zhao Y . NPY1, a BTB‐NPH3‐like protein, plays a critical role in auxin‐regulated organogenesis in Arabidopsis. Proc Natl Acad Sci USA. 2007;104(47):18825‐18829. 10.1073/pnas.0708506104 18000043 PMC2141861

[18] Bolton P , Macdonald H , Pickles A , et al. A case−control family history study of autism. J Child Psychol Psychiatry. 1994;35(5):877‐900. 10.1111/j.1469-7610.1994.tb02300.x 7962246

[19] Piven J , Gayle J , Chase GA , et al. A family history study of neuropsychiatric disorders in the adult siblings of autistic individuals. J Am Acad Child Adolesc Psychiatry. 1990;29(2):177‐183. 10.1097/00004583-199003000-00004 2324058

[20] Yin J , Schaaf CP . Autism genetics—an overview. Prenat Diagn. 2017;37(1):14‐30. 10.1002/pd.4942 27743394

[21] Sato W , Uono S . The atypical social brain network in autism: advances in structural and functional MRI studies. Curr Opin Neurol. 2019;32(4):617‐621. 10.1097/WCO.0000000000000713 31135458

[22] Boddaert N , Zilbovicius M , Philipe A , et al. MRI findings in 77 children with non‐syndromic autistic disorder. PLoS One. 2009;4(2):e4415. 10.1371/journal.pone.0004415 19204795 PMC2635956

[23] Hazlett HC , Gu H , Munsell BC , et al. Early brain development in infants at high risk for autism spectrum disorder. Nature. 2017;542(7641):348‐351. 10.1038/nature21369 28202961 PMC5336143

[24] Tang G , Gudsnuk K , Kuo SH , et al. Loss of mTOR‐dependent macroautophagy causes autistic‐like synaptic pruning deficits. Neuron. 2014;83(5):1131‐1143. 10.1016/j.neuron.2014.07.040 25155956 PMC4159743

[25] Bauman ML , Kemper TL . The neuropathology of the autism spectrum disorders: what have we learned? Novartis Found Symp. 2003;251:112‐122. 14521190

[26] Williams JH , Waiter GD , Gilchrist A , Perrett DI , Murray AD , Whiten A . Neural mechanisms of imitation and 'mirror neuron' functioning in autistic spectrum disorder. Neuropsychologia. 2006;44(4):610‐621. 10.1016/j.neuropsychologia.2005.06.010 16140346

[27] Kawicka A , Regulska‐Ilow B . How nutritional status, diet and dietary supplements can affect autism. A review. Rocz Panstw Zakl Hig. 2013;64(1):1‐12. 23789306

[28] Hyman SL , Stewart PA , Schmidt B , et al. Nutrient intake from food in children with autism. Pediatrics. 2012;130(Suppl 2):S145‐S153. 10.1542/peds.2012-0900L 23118245 PMC4536585

[29] Fujiwara T , Morisaki N , Honda Y , Sampei M , Tani Y . Chemicals, nutrition, and autism spectrum disorder: a mini‐review. Front Neurosci. 2016;10:174. 10.3389/fnins.2016.00174 27147957 PMC4837386

[30] Yasuda H , Yoshida K , Yasuda Y , Tsutsui T . Infantile zinc deficiency: association with autism spectrum disorders. Sci Rep. 2011;1:129. 10.1038/srep00129 22355646 PMC3216610

[31] Hagmeyer S , Sauer AK , Grabrucker AM . Prospects of zinc supplementation in autism spectrum disorders and shankopathies such as Phelan McDermid Syndrome. Front Synaptic Neurosci. 2018;10:11. 10.3389/fnsyn.2018.00011 29875651 PMC5974951

[32] Sun C , Zou M , Zhao D , Xia W , Wu L . Efficacy of folic acid supplementation in autistic children participating in structured teaching: an open‐label trial. Nutrients. 2016;8(6):337. 10.3390/nu8060337 27338456 PMC4924178

[33] Wiens D , DeSoto M . Is high folic acid intake a risk factor for autism?—a review. Brain Sci. 2017;7(12):149. 10.3390/brainsci7110149 29125540 PMC5704156

[34] Saad K , Abdel‐Rahman AA , Elserogy YM , et al. Vitamin D status in autism spectrum disorders and the efficacy of vitamin D supplementation in autistic children. Nutr Neurosci. 2016;19(8):346‐351. 10.1179/1476830515Y.0000000019 25876214

[35] Cannell JJ . Vitamin D and autism, what's new? Rev Endocr Metab Disord. 2017;18(2):183‐193. 10.1007/s11154-017-9409-0 28217829

[36] Krajcovicova‐Kudlackova M , Valachovicova M , Mislanova C , Hudecova Z , Sustrova M , Ostatnikova D . Plasma concentrations of selected antioxidants in autistic children and adolescents. Bratisl Lek Listy. 2009;110(4):247‐250. 19507654

[37] Parletta N , Niyonsenga T , Duff J . Omega‐3 and Omega‐6 polyunsaturated fatty acid levels and correlations with symptoms in children with attention deficit hyperactivity disorder, autistic spectrum disorder and typically developing controls. PLoS One. 2016;11(5):e0156432. 10.1371/journal.pone.0156432 27232999 PMC4883772

[38] Leader G , Tuohy E , Chen JL , Mannion A , Gilroy SP . Feeding problems, gastrointestinal symptoms, challenging behavior and sensory issues in children and adolescents with autism spectrum disorder. J Autism Dev Disord. 2020;50(4):1401‐1410. 10.1007/s10803-019-04357-7 31955310

[39] van De Sande MM , van Buul VJ , Brouns FJ . Autism and nutrition: the role of the gut−brain axis. Nutr Res Rev. 2014;27(2):199‐214. 10.1017/S0954422414000110 25004237

[40] Tognini P . Gut microbiota: a potential regulator of neurodevelopment. Front Cell Neurosci. 2017;11:25. 10.3389/fncel.2017.00025 28223922 PMC5293830

[41] Sanctuary MR , Kain JN , Angkustsiri K , German JB . Dietary considerations in autism spectrum disorders: the potential role of protein digestion and microbial putrefaction in the gut−brain axis. Front Nutr. 2018;5:40. 10.3389/fnut.2018.00040 29868601 PMC5968124

[42] Jyonouchi H . Food allergy and autism spectrum disorders: is there a link. Curr Allergy Asthma Rep. 2009;9(3):194‐201. 10.1007/s11882-009-0029-y 19348719

[43] Rossignol DA , Genuis SJ , Frye RE . Environmental toxicants and autism spectrum disorders: a systematic review. Transl Psychiatry. 2014;4(2):e360. 10.1038/tp.2014.4 24518398 PMC3944636

[44] Bent S , Hendren RL . Complementary and alternative treatments for autism part 1: evidence‐supported treatments. AMA J Ethics. 2015;17(4):369‐374. 10.1001/journalofethics.2015.17.4.sect1-1504 25901707

[45] Myers SM , Johnson CP , American Academy of Pediatrics Council on Children With Disabilities . Management of children with autism spectrum disorders. Pediatrics. 2007;120(5):1162‐1182. 10.1542/peds.2007-2362 17967921

[46] Coury DL , Anagnostou E , Manning‐Courtney P , et al. Use of psychotropic medication in children and adolescents with autism spectrum disorders. Pediatrics. 2012;130(Suppl 2):S69‐S76. 10.1542/peds.2012-0900D 23118256

[47] Casano AM , Peri F . Microglia: multitasking specialists of the brain. Dev Cell. 2015;32(4):469‐477. 10.1016/j.devcel.2015.01.018 25710533

[48] Bang M , Lee SH , Cho SH , et al. Herbal medicine treatment for children with autism spectrum disorder: a systematic review. Evid‐based Complement Alternat Med. 2017;2017:8614680. 10.1155/2017/8614680 28592982 PMC5448044

[49] Bertolino B , Crupi R , Impellizzeri D . Beneficial effects of co‐ultramicronized palmitoylethanolamide/luteolin in a mouse model of autism and in a case report of autism. CNS Neurosci Therapeut. 2017;23:87‐98. 10.1111/cns.12648 PMC6492645 27701827

[50] Banji D , Banji OJ , Abbagoni S , Hayath MS , Kambam S , Chiluka VL . Amelioration of behavioral aberrations and oxidative markers by green tea extract in valproate induced autism in animals. Brain Res. 2011;1410:141‐151. 10.1016/j.brainres.2011.06.063 21820650

[51] jayaprakash P , Isaev D , Shabbir W , Lorke DE , Sadek B , Oz M . Curcumin potentiates α7 nicotinic acetylcholine receptors and alleviates autistic‐like social deficits and brain oxidative stress status in mice. Int J Mol Sci. 2021;22(14):7251. 10.3390/ijms22147251 34298871 PMC8303708

[52] Zhong H , Xiao R , Ruan R , et al. Neonatal curcumin treatment restores hippocampal neurogenesis and improves autism‐related behaviors in a mouse model of autism. Psychopharmacology. 2020;237(12):3539‐3552. 10.1007/s00213-020-05634-5 32803366

[53] Pragnya B , Kameshwari JS , Veeresh B . Ameliorating effect of piperine on behavioral abnormalities and oxidative markers in sodium valproate induced autism in BALB/C mice. Behav Brain Res. 2014;270:86‐94. 10.1016/j.bbr.2014.04.045 24803211

[54] Sandhya T , Sowjanya J , Veeresh B . *Bacopa monniera* (L.) Wettst ameliorates behavioral alterations and oxidative markers in sodium valproate induced autism in rats. Neurochem Res. 2012;37(5):1121‐1131. 10.1007/s11064-012-0717-1 22322665

[55] Theoharides TC , Asadi S , Panagiotidou S . A case series of a luteolin formulation (NeuroProtek®) in children with autism spectrum disorders. Int J Immunopathol Pharmacol. 2012;25(2):317‐323. 10.1177/039463201202500201 22697063

[56] Tsilioni I , Taliou A , Francis K , Theoharides TC . Children with autism spectrum disorders, who improved with a luteolin‐containing dietary formulation, show reduced serum levels of TNF and IL‐6. Transl Psychiatry. 2015;5(9):e647. 10.1038/tp.2015.142 26418275 PMC5545641

[57] Taliou A , Zintzaras E , Lykouras L , Francis K . An open‐label pilot study of a formulation containing the anti‐inflammatory flavonoid luteolin and its effects on behavior in children with autism spectrum disorders. Clin Ther. 2013;35(5):592‐602. 10.1016/j.clinthera.2013.04.006 23688534

[58] Pretzsch CM , Voinescu B , Mendez MA , et al. The effect of cannabidiol (CBD) on low‐frequency activity and functional connectivity in the brain of adults with and without autism spectrum disorder (ASD). J Psychopharmacol. 2019;33(9):1141‐1148. 10.1177/0269881119858306 31237191 PMC6732821

[59] Aran A , Harel M , Cassuto H , et al Cannabinoid treatment for autism: a proof‐of‐concept randomized trial. Mol Autism. 2021;12:6. 10.1186/s13229-021-00420-2 33536055 PMC7860205

[60] Bashir S , Al‐Ayadhi LY . Effect of camel milk on thymus and activation‐regulated chemokine in autistic children: double‐blind study. Pediatr Res. 2014;75(4):559‐563. 10.1038/pr.2013.248 24375082

[61] Streit WJ , Mrak RE , Griffin WS . Microglia and neuroinflammation: a pathological perspective. J Neuroinflammation. 2004;1(1):14. 10.1186/1742-2094-1-14 15285801 PMC509427

[62] Kim JW , Hong JY , Bae SM . Microglia and autism spectrum disorder: overview of current evidence and novel immunomodulatory treatment options. Clin Psychopharmacol Neurosci. 2018;16(3):246‐252. 10.9758/cpn.2018.16.3.246 30121973 PMC6124874

[63] Gottfried C , Bambini‐Junior V , Francis F , Riesgo R , Savino W . The impact of neuroimmune alterations in autism spectrum disorder. Front Psychiatry. 2015;6:121. 10.3389/fpsyt.2015.00121 26441683 PMC4563148

[64] Marchezan J , Winkler Dos Santos E , Deckmann I , Riesgo R . Immunological dysfunction in autism spectrum disorder: a potential target for therapy. Neuroimmunomodulation. 2018;25(5‐6):300‐319. 10.1159/000492225 30184549

[65] Zuiki M , Chiyonobu T , Yoshida M , et al. Luteolin attenuates interleukin‐6‐mediated astrogliosis in human iPSC‐derived neural aggregates: a candidate preventive substance for maternal immune activation‐induced abnormalities. Neurosci Lett. 2017;653:296‐301. 10.1016/j.neulet.2017.06.004 28595950

[66] Xu N , Li X , Zhong Y . Inflammatory cytokines: potential biomarkers of immunologic dysfunction in autism spectrum disorders. Mediators Inflamm. 2015;2015:531518. 10.1155/2015/531518 25729218 PMC4333561

[67] Bahmani M , Sarrafchi A , Shirzad H , Rafieian‐Kopaei M . Autism: pathophysiology and promising herbal remedies. Curr Pharm Des. 2016;22(3):277‐285. 10.2174/1381612822666151112151529 26561063

[68] Bertolino B , Crupi R , Impellizzeri D , et al. Beneficial effects of co‐ultramicronized palmitoylethanolamide/luteolin in a mouse model of autism and in a case report of autism. CNS Neurosci Ther. 2017;23(1):87‐98. 10.1111/cns.12648 27701827 PMC6492645

[69] Chauhan A , Chauhan V . Oxidative stress in autism. Pathophysiology. 2006;13(3):171‐181. 10.1016/j.pathophys.2006.05.007 16766163

[70] Schimidt HL , Garcia A , Martins A , Mello‐Carpes PB , Carpes FP . Green tea supplementation produces better neuroprotective effects than red and black tea in Alzheimer‐like rat model. Food Res Int. 2017;100(Pt 1):442‐448. 10.1016/j.foodres.2017.07.026 28873707

[71] Cabrera C , Artacho R , Giménez R . Beneficial effects of green tea—a review. J Am Coll Nutr. 2006;25(2):79‐99. 10.1080/07315724.2006.10719518 16582024

[72] Takeda A , Sakamoto K , Tamano H , et al. Facilitated neurogenesis in the developing hippocampus after intake of theanine, an amino acid in tea leaves, and object recognition memory. Cell Mol Neurobiol. 2011;31(7):1079‐1088. 10.1007/s10571-011-9707-0 21604187 PMC11498479

[73] Sundberg M , Sahin M . Cerebellar development and autism spectrum disorder in tuberous sclerosis complex. J Child Neurol. 2015;30(14):1954‐1962. 10.1177/0883073815600870 26303409 PMC4644486

[74] Urdaneta KE , Castillo MA , Montiel N , Semprún‐Hernández N , Antonucci N , Siniscalco D . Autism spectrum disorders: potential neuro‐psychopharmacotherapeutic plant‐based drugs. Assay Drug Dev Technol. 2018;16(8):433‐444. 10.1089/adt.2018.848 30427697

[75] McNamara FN , Randall A , Gunthorpe MJ . Effects of piperine, the pungent component of black pepper, at the human vanilloid receptor (TRPV1). Br J Pharmacol. 2005;144(6):781‐790. 10.1038/sj.bjp.07060 15685214 PMC1576058

[76] Wattanathorn J , Chonpathompikunlert P , Muchimapura S , Priprem A , Tankamnerdthai O . Piperine, the potential functional food for mood and cognitive disorders. Food Chem Toxicol. 2008;46(9):3106‐3110. 10.1016/j.fct.2008.06.014 18639606

[77] Ornoy A , Weinstein‐Fudim L , Ergaz Z . Prevention or amelioration of autism‐like symptoms in animal models: will it bring us closer to treating human ASD. Int J Mol Sci. 2019;20(5):1074. 10.3390/ijms20051074 30832249 PMC6429371

[78] Ak T , Gülçin I . Antioxidant and radical scavenging properties of curcumin. Chem‐Biol Interact. 2008;174(1):27‐37. 10.1016/j.cbi.2008.05.003 18547552

[79] Cole GM , Teter B , Frautschy SA . Neuroprotective effects of curcumin. Adv Exp Med Biol. 2007;595:197‐212. 10.1007/978-0-387-46401-5_8 17569212 PMC2527619

[80] Al‐Askar M , Bhat RS , Selim M , Al‐Ayadhi L , El‐Ansary A . Postnatal treatment using curcumin supplements to amend the damage in VPA‐induced rodent models of autism. BMC Complement Altern Med. 2017;17(1):259. 10.1186/s12906-017-1763-7 28486989 PMC5424332

[81] Bhandari R , Kuhad A . Neuropsychopharmacotherapeutic efficacy of curcumin in experimental paradigm of autism spectrum disorders. Life Sci. 2015;141:156‐169. 10.1016/j.lfs.2015.09.012 26407474

[82] Panahi Y , Saadat A , Beiraghdar F , Sahebkar A . Adjuvant therapy with bioavailability‐boosted curcuminoids suppresses systemic inflammation and improves quality of life in patients with solid tumors: a randomized double‐blind placebo‐controlled trial. Phytother Res. 2014;28(10):1461‐1467. 10.1002/ptr.5149 24648302

[83] Solimini R , Rotolo MC , Pichini S , Pacifici R . Neurological disorders in medical use of cannabis: an update. CNS Neurol Disord Drug Targets. 2017;16(5):527‐533. 10.2174/1871527316666170413105421 28412919

[84] Shen LL , Jiang ML , Liu SS , et al. Curcumin improves synaptic plasticity impairment induced by HIV‐1gp120 V3 loop. Neural Regen Res. 2015;10(6):925‐931. 10.4103/1673-5374.158358 26199609 PMC4498354

[85] Salgado CA , Castellanos D . Autism spectrum disorder and cannabidiol: have we seen this movie before. Glob Pediatr Health. 2018;5:2333794X18815412. 10.1177/2333794X18815412 PMC6287295 30547057

[86] Nagarkatti P , Pandey R , Rieder SA , Hegde VL , Nagarkatti M . Cannabinoids as novel anti‐inflammatory drugs. Future Med Chem. 2009;1(7):1333‐1349. 10.4155/fmc.09.93 20191092 PMC2828614

[87] Perucca E . Cannabinoids in the treatment of epilepsy: hard evidence at last? J Epilepsy Res. 2017;7(2):61‐76. 10.14581/jer.17012 29344464 PMC5767492

[88] Zamberletti E , Gabaglio M , Parolaro D . The endocannabinoid system and autism spectrum disorders: insights from animal models. Int J Mol Sci. 2017;18(9):1916. 10.3390/ijms18091916 28880200 PMC5618565

[89] Brigida AL , Schultz S , Cascone M , Antonucci N , Siniscalco D . Endocannabinod signal dysregulation in autism spectrum disorders: a correlation link between inflammatory state and neuro‐immune alterations. Int J Mol Sci. 2017;18(7):1425. 10.3390/ijms18071425 28671614 PMC5535916

[90] Ude C , Schubert‐Zsilavecz M , Wurglics M . *Ginkgo biloba* extracts: a review of the pharmacokinetics of the active ingredients. Clin Pharmacokinet. 2013;52(9):727‐749. 10.1007/s40262-013-0074-5 23703577

[91] Fang W , Deng Y , Li Y , et al. Blood brain barrier permeability and therapeutic time window of Ginkgolide B in ischemia‐reperfusion injury. Eur J Pharmaceut Sci. 2010;39(1‐3):8‐14. 10.1016/j.ejps.2009.10.002 19833202

[92] Niederhofer H . First preliminary results of an observation of *Ginkgo biloba* treating patients with autistic disorder. Phytother Res. 2009;23(11):1645‐1646. 10.1002/ptr.2778 19274699

[93] Hasanzadeh E , Mohammadi MR , Ghanizadeh A , et al. A double‐blind placebo controlled trial of *Ginkgo biloba* added to risperidone in patients with autistic disorders. Child Psychiatry Hum Dev. 2012;43(5):674‐682. 10.1007/s10578-012-0292-3 22392415

[94] Malishev R , Shaham‐Niv S , Nandi S , Kolusheva S , Gazit E , Jelinek R . Bacoside‐A, an Indian traditional‐medicine substance, inhibits β‐Amyloid cytotoxicity, fibrillation, and membrane interactions. ACS Chem Neurosci. 2017;8(4):884‐891. 10.1021/acschemneuro.6b00438 28094495

[95] Russo A , Borrelli F . *Bacopa monniera*, a reputed nootropic plant: an overview. Phytomedicine. 2005;12(4):305‐317. 10.1016/j.phymed.2003.12.008 15898709

[96] Hoffman EJ , Turner KJ , Fernandez JM , et al. Estrogens suppress a behavioral phenotype in Zebrafish mutants of the autism risk gene, CNTNAP2. Neuron. 2016;89(4):725‐733. 10.1016/j.neuron.2015.12.039 26833134 PMC4766582

[97] Al‐Ayadhi LY , Elamin NE . Camel milk as a potential therapy as an antioxidant in autism spectrum disorder (ASD). Evid‐Based Complement Alternat Med. 2013;2013:602834. 10.1155/2013/602834 24069051 PMC3773435

[98] Kappeler S , Farah Z , Puhan Z . Sequence analysis of *Camelus dromedarius* milk caseins. J Dairy Res. 1998;65(2):209‐222. 10.1017/s0022029997002847 9627840

[99] Zafra O , Fraile S , Gutiérrez C , et al. Monitoring biodegradative enzymes with nanobodies raised in *Camelus dromedarius* with mixtures of catabolic proteins. Environ Microbiol. 2011;13(4):960‐974. 10.1111/j.1462-2920.2010.02401.x 21219561

[100] Shabo Y , Barzel R , Margoulis M , Yagil R . Camel milk for food allergies in children. Isr Med Assoc J. 2005;7(12):796‐798. 16382703

[101] Tillib SV , Ivanova TI , Vasilev LA . Fingerprint‐like analysis of “nanoantibody” selection by phage display using two helper phage variants. Acta Naturae. 2010;2(3):85‐93. 22649655 PMC3347569

